# Prevalence, correlates and treatment needs of dental caries among people on antiretroviral therapy in Uganda: a cross sectional study

**DOI:** 10.1186/s12903-022-02256-5

**Published:** 2022-06-10

**Authors:** Wilfred Arubaku, Godfrey Kwizera, Deusdedit Tusubira, Michael Kanyesigye, Steffany Chamut, Brittany Anne Seymour, Mark J. Siedner, Vallence Niyonzima, Josephine N. Najjuma, Samuel Maling

**Affiliations:** 1 Department of Dental Surgery, Mbarara University of Science and Technology, Mbarara, Uganda; 2 Department of Biochemistry, Mbarara University of Science and Technology, Mbarara, Uganda; 3 Mbarara Regional Referral Hospital, Mbarara, Uganda; 4 Department of Oral Health Policy and Epidemiology, Harvard School of Dental Medicine, Boston, USA; 5 Department of Medicine, Massachusetts General Hospital, Boston, USA; 6 Department of Medicine, Harvard Medical School, Boston, USA; 7 Department of Medicine, Mbarara University of Science and Technology, Mbarara, Uganda; 8 Department of Nursing, Mbarara University of Science and Technology, Mbarara, Uganda; 9 Department of Psychiatry, Mbarara University of Science and Technology, P O Box 1410, Mbarara, Uganda

**Keywords:** Dental caries, Antiretroviral therapy, HIV, Oral diseases

## Abstract

**Background:**

Oral diseases are estimated to affect half of people living with HIV in the western world, and are often marked by pain, discomfort, disfigurement and reduced quality of life. Both HIV-specific and antiretroviral therapy-associated oral diseases have been found in this population. However, the prevalence, correlates and treatment needs of dental caries among people on antiretroviral therapy has not been well studied in rural Africa where majority of people living with HIV reside. Moreover, health behaviors and access to dental care vary significantly from high-income countries in the global north.

**Methods:**

A cross-sectional study was conducted among people living with HIV attending a high-volume HIV clinic with an enrollment of 10,000 patients in a regional referral hospital in Southwestern Uganda. The clinic is located in an urban setting with a large rural catchment area. Oral health data was collected using the modified World Health Organization oral health questionnaire for adults. Dental examinations were conducted to identify and classify dental caries using the decayed, missing, filled, teeth (DMFT) index and compute the treatment need. Logistic regression models were employed to identify correlate of dental caries.

**Results:**

A total of 194 participants were included in the study. The majority were female (124/194, 63.9%) with a median age of 42 years (IQR 36–49). The prevalence of dental caries experience among study participants was 67%, (130/194, 95% CI 60–75%). The mean DMFT index score was 4 (IQR 2–6) and treatment need was 96% (192/200). A higher CD4 count was associated with the presence of dental caries (OR 0.403, 95% CI 0.175–0.932) although it was not significant in multivariate analysis.

**Conclusion:**

There is a high prevalence of dental caries among people living with HIV on ART in Uganda. Our data demonstrate a high oral treatment need among this population. We recommend inclusion of preventive and therapeutic oral care into HIV care in this region.

## Background

Globally, oral diseases affect nearly 3.5 billion people [1]. Untreated dental caries in permanent teeth is the most prevalent and common oral health condition [2]. Evidence has shown that oral diseases affect 40–50% of people living with HIV (PLWH) and often begin early in the disease's course [3]. Oral diseases frequently result in pain, discomfort, deformity, and a reduction in quality of life. Furthermore, they reduce the productivity of those afflicted, delaying the fulfillment of Sustainable Development Goals 1 and 2 [4]. HIV increases the risk of immune-mediated oral diseases and complications [5]. On the other hand, the long-term side effects of Antiretroviral Therapy also include oral diseases and complications [6, 7]. Uganda continues to have a high burden of HIV with a prevalence of 6.2% among adults; 7.6% among women and 4.7% among men [8]. Among people living with HIV in Uganda, 65.5% are on antiretroviral therapy (ART) [8]. Although ART has improved the life expectancy among people living with HIV, more are prone to non-communicable diseases including dental caries [9, 10]. HIV infection and duration of ART use have been found to influence salivary flow and its buffering effects among adults and predisposing them to development of dental caries [6, 11]. Oral and dental health remains a neglected area in most national and global health policy frameworks including HIV care; thus, contributing less than optimal overall health outcomes in this category of patients.

In Uganda, a national wide oral health survey report (2015) found a high burden of dental caries experience, with a prevalence of 66% among the adult population [12]. Multiple other studies in Uganda have reported a prevalence of dental caries over 50% [13]. Dental caries have also been found to be prevalent specifically among PLWH, and have been associated with ART use [11, 14]. For example, Kalanzi [6] reported a prevalence of dental caries of 84% in an urban population of adults living with HIV. ART use has been associated with severe forms of dental caries [15] and has been implicated in pyogenic odontogenic infections [16] particularly in individuals with compromised immunity.

In summary, current available literature has demonstrated high prevalence of dental caries among PLWH and ART use in urban populations of Uganda [6]. However, there is scanty data from rural settings in Uganda, where majority of Ugandans reside and access to dental care is limited. The aim of this study was to determine the prevalence of dental caries among PLWH attending an adult outpatients HIV clinic in rural south-western Uganda.

## Methods

### Study design and setting

A cross-sectional study was conducted during May–June in 2017 at the adult HIV clinic of Mbarara Regional Referral Hospital (MRRH) located in Mbarara city, 275 km southwest of the capital city, Kampala. The study was done as part of an oral health status assessment of PLWH in rural south western Uganda. The MRRH HIV clinic has an enrollment of over 10,000 patients, and initiates approximately 1000 new patients on antiretroviral therapy (ART) each year. MRRH serves a catchment population of over 8,000,000 people drawn from 17 districts of rural Southwestern Uganda.

### Ethical considerations

All participants provided written informed consent to participate in the study. A separate consent to review participants’ clinical records was also obtained. For participants who were not formally educated and unable to read and write, informed consent was read to them verbatim in the local language of their preference. When they agreed to participate in the study, they were asked to append their thumb print to the informed consent form and the translator signed as a witness. The Mbarara University Research Ethics Committee (MUST-REC) reviewed and approved the proposal (Reference number: 17/02-17). Participants with dental treatment needs detected by this study were referred to the dental unit at MRRH for management.

### Inclusion criteria and recruitment

We selectively recruited adult patients aged 18 years, and older receiving care at MRRH HIV clinic, and either taking or initiating ART. The selection was performed following a systematic sampling approach in which every fifth patient attending the clinic who met these criteria was included in the study. If a patient declined treatment or failed to meet the inclusion criteria, the subsequent patient was considered.

### Sample size

We determined the sample size of 194 participants using the Krejcie and Morgan table [17] basing on an average of 380 clients attending the adult HIV clinic per month assuming population proportion of 0.5 and confidence interval of 95%.

### Data collection

An interviewer administered questionnaire with structured questions to collect data was used. Socio-demographic and clinical data were collected including age, gender, ART use and history, smoking history, alcohol use, brushing practices and dental caries using a modified World Health Organization (WHO) oral health questionnaire for adults [18]. The questionnaire was administered by trained research assistants.

Dental examinations were carried out by a trained and calibrated dental surgeon using disposable dental mirrors and probes under natural light. First, the number of missing teeth was noted, and surfaces of all teeth present were inspected for the presence or absence of dental caries, with or without fillings; and findings recorded on a dental chart. Caries experience were further classified using the decayed, missing, filled, teeth (DMFT) index [19]. Teeth that were traumatized or malformed, missing naturally, or extracted after trauma, existing periodontal disease or surgical intervention involving the mouth were excluded from classification. Those teeth which are filled because of dental caries were defined as having caries, whereas those restored following trauma or for cosmetic purposes were not. The respondents with toothache or dental caries were referred to the dental clinic for management, while those with missing teeth were advised to have dentures if necessary according to standard care of the Uganda Ministry of Health. All guidelines by the Uganda National Council of Science and Technology for conducting human participants research were fully adhered to.

### Treatment needs assessment

We computed treatment needs by adding participants who had decayed and missing teeth and dividing by the total number of participants who had decayed, missing and filled teeth as previously used by Aleksejūnienė and Vilma Brukienė [20].

### Statistical methods

We conducted descriptive analyses on socio-demographic, oral health, and HIV characteristics of the cohort, then stratified by the presence or absence of dental caries. Estimated crude prevalence of dental caries and summarized DMFT index were obtained. Using fitted logistic regression models we identified correlates of the presence of dental caries. Our predictor variables of interest included socio-demographic factors (age, sex, education and income, which was categorized based on an income greater than vs less than $1/day [21]), alcohol and tobacco use, dental health practices (number of times meals were eaten, frequency of brushing and dental checkups), dietary habits (eating snacks between meals) and HIV medical history (ART use, current CD4 count and CD4 at the time of initiation of ART). Variables with significance of *p* < 0.25 in univariable models were included in multivariable models. Analyses were conducted with STATA version 13 (Statacorp, College Station, TX).

## Results

### Participant characteristics

We enrolled 194 participants, with a median age of 42 (IQR 36–49) years. The majority was female (64%, 124/194). The median ART duration was 8 years (IQR 8–12) for the total cohort, and for those with and without dental caries experience was 7 (IQR 4–12) and 9 years (IQR 3.5–12) respectively. A majority of participants had CD4 count more than 350cells/µl (78%, 152/194). Most participants reported having two meals per day (54%, 105/194) and brushing their teeth using either a toothbrush or chew stick at least once a day (86%, 167/194). Most of our participants did not smoke (93%, 180/194) nor drink (80%, 156/194). The majority (78%, 152/194) engaged in snacking and most (91%, 176/194) had never visited a dentist for dental checkup (see Table 1). The median ART duration in years for participants with no dental caries experience was 9 (IQR 3.5–12) and those with caries was 7 (IQR 4–12) with a non-significant *P* value of 0.307.

**Table 1:** Characteristics of participants according to presence or absence of dental caries

Characteristic	No dental caries	Dental caries	*P* value
	(N = 64)	(N = 130)	
	n (%)	n (%)	
*Age categories*			
< 36	12 (18.8)	35 (26.9)	0.29
36–45	25 (39.1)	46 (35.4)	
> 45	27 (42.2)	49 (37.7)	
*Gender*			
Female	39 (60.9)	85 (65.4)	0.544
Male	25 (39.1)	45 (34.6)	
*Education*			
None	9 (14.1)	16 (12.3)	
Primary	39 (60.9)	66 (50.8)	0.293
Secondary	10 (15.6)	33 (25.4)	
Certificate	3 (4.7)	10 (7.7)	
Diploma	3 (4.7)	5 (3.9)	
*Occupation*			
Formal employment	5 (7.8)	15 (11.5)	
Business	6 (9.4)	9 (6.9)	0.591
Casual worker	34 (53.1)	51 (39.2)	
Self employed	19 (29.7)	55 (42.3)	
*CD4 count*			
≤ 350	8 (12.5)	34 (26.2)	0.034^*^
> 350	56 (87.5)	96 (73.8)	
*Meals per day*			
Once	6 (9.4)	12 (9.2)	
Twice	38 (59.4)	67 (51.5)	0.665
Thrice	18 (28.1)	48 (36.9)	
> 3 times	2 (3.1)	3 (2.3)	
*Tooth brushing habits*			
No	13 (20.3)	14 (10.8)	
At least once a day	51 (79.7)	116 (89.2)	0.075
*Alcohol use*			
No	49 (76.6)	107 (82.3)	0.343
Yes	15 (23.4)	23 (17.7)	
*Smoking*			
No	59 (92.2)	121 (93.1)	0.822
Yes	5 (7.8)	9 (6.9)	
*Ever had a dental checkup*			
Never	58 (90.6)	118 (90.8)	0.903
Once	6 (9.4)	10 (7.7)	
Twice	0 (0)	1 (0.8)	
3 + times	0 (0)	1 (0.8)	
*Eating snacks*			
No	12 (18.7)	30 (23.1)	
Yes	52 (81.3)	100 (76.9)	0.491
*Use of other drugs*			
No	59 (92.2)	113 (86.9)	0.277
Yes	5 (7.8)	17 (13.1)	

*P* values represent tests for differences in characteristics for people with and without carries experience and were calculated using chi-squared testing

n = participants

### Prevalence of carries and oral treatment needs

We found about two thirds of the participants (67%, 130/194) (95% CI 0.603–0.737) had dental caries using a DMFT > 0 (see Table 2). The median DMFT index score was 4 (IQR 2–6). The decayed component was 113 (median 2, IQR 2–3), missing 79 (median 2, IQR 1–4), filled 8 (median 2.5, IQR 1–3.5). The median ART duration in years for participants with decayed teeth was 7.0 (IQR 0.4–11), missing was 7.0 (IQR 4–12), and those with filled teeth was 8 (IQR 0.5–11.5). The ART duration was not statistically significant between participants with decayed, missing and filled teeth.

**Table 2:** DMFT and oral treatment needs among people living with HIV in southwestern Uganda

Characteristic	Decayed	Missing	Filled	Mean DMFT*		Treatment need**		*P*-value***	
	(N = 113)	(N = 79)	(N = 8)						
	n (%)	n (%)	n (%)			%			
*Age categories*									
< 36	31 (27.4)	18 (22.8)	4 (50)	3		96		0.404	
36–45	42 (37.2)	30 (38.0)	2 (25)	3.4		99.4			
> 45	40 (35.4)	31 (39.2)	2 (25)	2.6		98.5			
*Gender*									
Male	35 (31.0)	27 (34.2)	1 (12.5)	2.4		98.7		0.121	
Female	78 (69.0)	52 (65.8)	7 (87.5)	3.3		97.9			
*Education*									
None	16 (14.2)	9 (11.4)	2 (25.0)	2.8		100		0.927	
Primary	58 (51.3)	38 (48.1)	0 (0)	3		99.1			
Secondary	28 (24.8)	22 (27.9)	3 (37.5)	3.4		97.6			
Certificate	7 (6.2)	7 (8.9)	1 (12.5)	2.6		96			
Diploma	4 (3.5)	3 (3.8)	2 (25.0)	2.4		88.2			
*Occupation*									
Formal Employment	14 (12.39)	7 (8.9)	2 (25.0)	2.6		97.2		0.813	
Business	7 (6.2)	4 (5.1)	1 (12.5)	2.4		100			
unskilled worker	46 (40.7)	33 (41.8)	1 (12.5)	3		99			
Self employed	46 (40.7)	35 (44.)	4 (50.0)	3.2		97.4			
*CD4 count*									
≤ 350	27 (23.9)	21 (26.6)	2 (25.0)	3.3		99.2		0.57	
> 350	86 (76.1)	58 (73.4)	67 (75.0)	2.9		97.8			
*Meals per day*									
Once	10 (8.9)	8 (10.1)	4 (50.0)	3.2		100		0.946	
Twice	57 (50.4)	42 (53.2)	0 (0)	2.9		99.1			
Thrice	44 (38.9)	26 (32.9)	3 (37.5)	3.1		97.4			
> 3 times	2 (1.8)	3 (3.8)	1 (12.5)	3.8		83.3			
*Tooth brushing habits*									
No	13 (11.5)	6 (7.6)	0 (0)	1.9		100		0.093	
At least once a day	100 (88.5)	73 (92.4)	8 (100)	3.2		98			
*Alcohol use*									
No	95 (84.1)	65 (82.3)	7 (87.5)	3.1		97.9		0.51	
Yes	18 (15.9)	14 (17.7)	1 (12.5)	2.7		99.6			
*Smoking*									
No	106 (93.8)	74 (93.7)	8 (100)	3.1		98		0.354	
Yes	7 (6.2)	5 (6.3)	0 (0)	2.1		100			
*Ever had a dental checkup*									
Never	104 (92.0)	68 (86.1)	6 (75.0)	3		98.5		0.169	
Once	7 (6.2)	9 (11.4)	1 (12.5)	2.9		98.3			
Twice	1 (0.9)	1 (1.3)	0 (0)			100			
3+ times	1 (0.9)	1 (1.3)	1 (12.5)			60			
*Eating snacks*									
No	26 (23.0)	16 (20.3)	0 (0)	2.8		100		0.728	
Yes	87 (77.0)	63 (79.7)	8 (100)	3.1		97.6			
*Use of other drugs*									
No	101 (89.4)	70 (88.6)	7 (87.5)	3.1		97.9		0.231	
Yes	12 (10.6)	9 (11.4)	1 (12.5)	2.1		100			

*DMFT, decayed missing, and filled teeth

**Treatment need (decayed + missing teeth/decayed + missing + filled teeth) %

***p-values of significance between mean DMFT according to social participant characteristics calculated using chi-square testing

n = participants

In bivariate analysis, CD4 count was associated with the presence of dental carries (OR = 0.4 CI 0.175–0.932, *P* value 0.034) meanwhile in multivariate analysis there were no significant results (see Table 3).

**Table 3:** Logistic regression models for correlates of dental carries among people with HIV

Characteristics	Bi-variate analysis			Multivariate analysis		
	OR	CI	*P* value	AOR	CI	*P* values
Age	0.987	0.957–1.017	0.399	0.982	0.947–1.020	0.362
Gender	0.826	0.445–1.533	0.544	0.852	0.413–1.757	0.664
Education	1.196	0.856–1.671	0.293	1.269	0.845–1.907	0.250
Occupation	1.091	0.793–1.501	0.591	1.235	0.863–1.769	0.248
ART	0.816	0.320–2.081	0.671	1.104	0.330–3.690	0.872
ART duration	0.971	0.919–1.027	0.307	0.987	0.916–1.062	0.725
CD4 count	0.403	0.175–0.932	0.034*	0.427	0.172–1.063	0.067
Meals per day	1.179	0.751–1.849	0.475	1.098	0.665–1.811	0.715
Tooth brushing habits	2.112	0.927–4.813	0.075	1.811	0.748–4.384	0.188
Income	1.0	1–1	0.267	1.0	1.0–1.0	0.148
Alcohol use	0.702	0.337–1.462	0.345	0.614	0.255–1.478	0.277
Smoking	0.878	0.282–2.735	0.822	1.190	0.294–4.809	0.807
Dental checkup	1.179	0.504–2.761	0.704	0.995	0.409–2.425	0.992
Eating snacks	0.769	0.364–1.626	0.492	0.759	0.332–1.732	0.512
Use of other drugs	1.775	0.624–5.051	0.282	1.960	0.587–6.550	0.274

*CD4 count was significantly associated with dental caries

## Discussion

Our study estimated the prevalence, correlates and treatment needs of dental caries among PLWH in rural Uganda. Our findings highlight the high prevalence of carries (67%) among this population, with the decayed component among those with dental caries experience also being very high (113/130, 86.9%). Moreover, we identified an association between a higher CD4 count (> 350 cells/uL) with lower odds (0.4) of dental caries. The results also reveal a gap in oral health prevention needs, with over 90% of participants never having had a dental evaluation, despite having dental caries. Finally, the gap between the decayed teeth (DT) 113 and filled teeth (FT) (8) is a clear demonstration of possible inadequacies in knowledge, availability or access to dental services that require further investigations.

Our findings of a high prevalence of dental carries is similar to what was found in an urban population in Uganda on ART [6], in which the majority of participants (65.6%) were female and were young with mean age of 39 years. In another study on women living with HIV and on ART carried out in Eastern Uganda, those who had dental caries, were also young with mean age of 35 years [22]. Two thirds of our study participants (67%) had dental caries (DMFT > 0). Although there was a higher overall prevalence of dental caries in women (n = 85, 65.4%) compared to men (n = 45, 34.6%) the difference was not statistically significant (*P* value 0.544). This is similar to those found by Kalanzi et al. (2019) who found a high overall prevalence of 83.7% and a higher prevalence among females 86.6% than males 78.2% [6]. Birungi and others (2021) also found a high prevalence of dental caries (81%) in a rural population of HIV positive women [22]. The reasons as to why women in this study population have a higher prevalence of dental caries than men are not clear but may be due to differences in snacking, oral hygiene and dietary habits thus a need for further research in this area. The high prevalence of dental caries in our study is a reflection of the general population probably due to lack of preventive and curatives oral services. In the study setting, there is no integration of oral services with HIV care thus the unmet prevention and treatment need for oral health.

In our regression model the OR is 0.4 for CD4 count suggests a lower odd with higher CD4 count in this group. This is similar to other studies that suggest a lower CD4 count being associated with higher dental caries experience [6, 23]. However, others have found no association between CD4 count and dental caries [24]. Therefore, this is an area that requires further exploration to investigate the conflicting role of ART in dental caries.

Our findings reveal a large prevention need as demonstrated by a high proportion of participants who have never gone for a checkup (n = 176, 90.7%) of these 90.8% had caries. Furthermore, we found a high overall oral treatment need of 96% as the number of patients with decayed and/or missing teeth had never had them filled or replaced. Additionally, 13.9% reported never brushed their teeth at all this call for need for oral health education intervention. Among participants with dental caries experience (DMFT < 0) 86% (113/130) had decayed teeth which were not filled and 60% (79/130) had missing teeth which were not replaced. This further raises a need for a large-scale survey among people on ART to generate stronger evidence for integrating dental and oral health into HIV care. Besides the evidence from a low filled 6% (8/130) component of DMFT index despite a high decay 86% (113/130) component is strong indication for lack of individual dental care for the patient as decaying teeth are ignored. The high proportion of decayed but not filled and missing and not replaced teeth demonstrates a significant unmeet need for oral healthcare in this setting. To address this gap policy makers could consider integrating oral health services within HIV clinics, to provide greater access to oral health education and care. Additionally, to mitigate this glaring disparity, there is a need to include dental and oral health care services in the national guidelines for HIV care.

The limitations of our study include the fact that this work was conducted in a high-volume clinic in a single regional referral hospital, the cross-sectional nature of the study that prevents determination of causality, and the lack of an HIV uninfected comparator group to explore HIV-specific effects. Future work should include longitudinal follow-up and a non-HIV comparison group to account for these limitations.

## Conclusions

We identified a high prevalence and treatment needs of dental caries in PLWH on ART in rural southwestern Uganda, where there is an absence of preventive and therapeutic care for oral health. Consequently, we recommend inclusion of dental and oral care in routine HIV care services in the region.

## Data Availability

All data generated or analysed during this study are included in this published article.

## References

[1] Marcenes W, Kassebaum NJ, Bernabé E, Flaxman A, Naghavi M, Lopez A, Murray CJ (2013). Global burden of oral conditions in 1990–2010: a systematic analysis. J Dent Res.

[2] World Health Organisation (2021). Oral health fact sheet.

[3] Dye B (2017). The global burden of oral disease: research and public health significance. J Dent Res.

[4] United Nations. Sustainable development goals. *The energy progress report Tracking SDG* 2019;7.

[5] Khoury ZH, Meeks V (2021). The influence of antiretroviral therapy on HIV-related oral manifestations. J Natl Med Assoc.

[6] Kalanzi D, Mayanja-Kizza H, Nakanjako D, Mwesigwa CL, Ssenyonga R, Amaechi BT (2019). Prevalence and factors associated with dental caries in patients attending an HIV care clinic in Uganda: a cross sectional study. BMC Oral Health.

[7] Nittayananta W, Talungchit S, Jaruratanasirikul S, Silpapojakul K, Chayakul P, Nilmanat A, Pruphetkaew N (2010). Effects of long-term use of HAART on oral health status of HIV-infected subjects. J Oral Pathol Med Off Publ Int Assoc Oral Pathol Am Acad Oral Pathol.

[8] Ministry of Health Uganda (2017). Uganda population-based HIV impact assessment.

[9] Achwoka D, Waruru A, Chen T-H, Masamaro K, Ngugi E, Kimani M, Mukui I, Oyugi JO, Mutave R, Achia T (2019). Noncommunicable disease burden among HIV patients in care: a national retrospective longitudinal analysis of HIV-treatment outcomes in Kenya, 2003–2013. BMC Public Health.

[10] Wandeler G, Johnson LF, Egger M (2016). Trends in life expectancy of HIV-positive adults on antiretroviral therapy across the globe: comparisons with general population. Curr Opin HIV AIDS.

[11] Cavasin Filho JC, Giovani EM (2009). Xerostomy, dental caries and periodontal disease in HIV+ patients. Braz J Infect Dis Off Publ Braz Soc Infect Dis.

[12] Kutesa A, Kasangaki A, Nkamba M, Muwazi L, Okullo I, Rwenyonyi CM (2015). Prevalence and factors associated with dental caries among children and adults in selected districts in Uganda. Afr Health Sci.

[13] Rwenyonyi CM, Muwazi LM, Buwembo W (2011). Assessment of factors associated with dental caries in rural communities in Rakai District, Uganda. Clin Oral Investig.

[14] Rezaei-Soufi L, Davoodi P, Jazaeri M, Niknami H (2011). The comparison of root caries experience between HIV-positive patients and HIV-negative individuals in a selected Iranian population. Int J Dent Hyg.

[15] Kalanzi D, Mayanja-Kizza H, Nakanjako D, Sewankambo NK (2018). Extensive dental caries in a HIV positive adult patient on ART; case report and literature review. BMC Oral Health.

[16] Kityamuwesi R, Muwaz L, Kasangaki A, Kajumbula H, Rwenyonyi CM (2015). Characteristics of pyogenic odontogenic infection in patients attending Mulago Hospital, Uganda: a cross-sectional study. BMC Microbiol.

[17] Krejcie RV, Morgan DW (1970). Determining sample size for research activities. Educ Psychol Meas.

[18] World Health Organization (2013). Oral health surveys: basic methods.

[19] World Health Organization (2013). Oral health survey: basic methods.

[20] Aleksejūnienė J, Brukienė V (2009). An assessment of dental treatment need: an overview of available methods and suggestions for a new, comparative summative index. J Public Health Dent.

[21] Owori M. Poverty in Uganda: National and regional data and trends. Development Initiatives. https://devinit.org/resources/poverty-uganda-national-and-regional-data-and-trends. 2020.

[22] Birungi N, Fadnes LT, Engebretsen IMS, Tumwine JK, Lie SA, Åstrøm AN (2021). Caries experience by socio-behavioural characteristics in HIV-1-infected and uninfected Ugandan mothers: a multilevel analysis. Acta Odontol Scand.

[23] Kasiraj G (2012). Correlation of CD4 count with dental caries in HIV seropositive individuals with and without ART (Antiretroviral Therapy). Open Access Sci Rep.

[24] Souza AJ, Gomes-Filho IS, Silva C, Passos-Soares JS, Cruz SSD, Trindade SC, Figueiredo A, Buischi YP, Seymour GJ, Cerqueira EMM (2018). Factors associated with dental caries, periodontitis and intra-oral lesions in individuals with HIV/AIDS. AIDS Care.

